# Nurturing and Optimizing Networks of Care to Maximize Benefits to Patients, Health Workers, and Health Systems

**DOI:** 10.9745/GHSP-D-22-00516

**Published:** 2022-12-21

**Authors:** Kelly Saldaña, Nicholas Leydon, Steve Hodgins

**Affiliations:** a Abt Associates, Washington, DC, USA.; b Bill & Melinda Gates Foundation, Seattle, WA, USA.; c Editor-in-Chief, Global Health: Science and Practice Journal; School of Public Health, University of Alberta, Edmonton, Canada.

See related article by Kalaris et al.

In a commentary summarizing a landscape review of Networks of Care conducted by the World Health Organization, Kalaris et al.^1^ argue that Networks of Care can improve quality and continuity of care and strengthen health systems functions by improving relationships between different providers—and that all these improvements will accelerate progress toward the Sustainable Development Goals.

This is a lot of hope to place on one approach. Often in global health, promising practices such as these are oversimplified and oversold as a panacea, ultimately underdelivering on their promise of success. Although we share the authors’ optimism regarding Networks of Care, we think that to ensure that Networks of Care ultimately lead to more effective, replicable patient care, global health practitioners must galvanize more implementation research, operations management capacities, and facilitative policies that elucidate intrinsic and extrinsic factors influencing and unlocking the potential of Networks of Care.

## POTENTIAL AVENUES

Building a solid evidence base for Networks of Care first requires a clear understanding of the problems such networks could solve. Evidence generation can then focus on the extent to which they have achieved their intended benefits, which may vary by circumstances. Evidence needed includes documentation of the benefits accrued through existing and informal Networks of Care, what specific incentive structures have helped (such as payments to traditional birth attendants for referring obstetric patients to more formal care providers), and what adaptation of these incentives could further strengthen network function.

There are 5 areas where Networks of Care may be a potential solution:

1. **New approaches are needed to improve quality of care.** Gaps in quality of care are now responsible for more deaths globally than inadequate access.^2^ While better infrastructure and staffing have improved key measures for many countries, the next frontier is service-level improvement, including revisions to existing organization of services. While promising, the process of establishing new countrywide service delivery models takes time and political will; Networks of Care offer a more immediate opportunity to address structural aspects of quality. Furthermore, Networks of Care have a potentially important role in ensuring quality of care, which can span multiple episodes of care, different service providers, and changing standards of practice.
2. **Our understanding of quality needs to be centered on the patient.** Patient outcomes are the bottom-line measure of quality. Patients will seek care from the options available based on what they feel best meets their needs in terms of convenience and the specific care needed for the condition they are facing. They may seek care from the public sector, private sector, drug shops, or other informal providers. They may enter at different points in the system, from primary to tertiary level. Patients are influenced in these choices by their communities and local context—prioritizing care that they trust and can access—and by providers they feel are respectful. Networks of Care can help ensure that standards of quality are met, ensure that local voices are included in care constructs, and support ongoing patient contact as they make different care calculations to meet their evolving needs.
3. **Changing disease profiles complicate care.** Many low- and middle-income countries now simultaneously work to improve traditional health programs, such as safe deliveries, family planning, and immunizations, alongside more recently adopted service priorities, such as noncommunicable diseases and mental health services. Although the global health community has tended to address each of these as standalone programs, from an individual’s perspective, this increasingly complex set of services and patient needs guarantees that no health care provider is fully equipped to address all the needs of an individual or family. Networks of Care can help ensure better continuity of care by strengthening handoffs between providers, including among those offering more specialized services, as needed.
4. **Health workers need to be viewed not only as professionals but also as human beings.** Faced with compounding workload burdens, shortages of supplies, lack of supervision and effective management, and high patient loads, health workers at all levels benefit from peer networks and trusted mentorship for problem solving.^3^ When nurtured properly, Networks of Care can help health workers collaborate to solve problems, learn lessons from each other, and commiserate on common issues. Such support increases job satisfaction, interprofessional respect, and, ultimately, worker retention. More sophisticated Networks of Care can evolve to support professional development, accreditation, and advocacy efforts.
5. **Crises require coordination which, in turn, requires functional relationships.** Because emergencies often demand clear, urgent communication, responders benefit from well-worn methods for engaging trusted partners. This is practiced through ongoing coordination of patient care and collective problem solving in the face of “everyday challenges,” such as stock-outs of drugs or supplies, diagnostic equipment breakdowns, or worker shortages in 1 facility that are covered through network and partnerships with other facilities. In addition to these everyday challenges, countries will continue to experience shocks, including droughts, floods, disease outbreaks, and armed conflict. Health systems can blunt these shocks and recover more quickly when care providers communicate effectively. Such communication is bolstered through relationships developed through Networks of Care.

## CLEAR BENEFITS

Ultimately, Networks of Care will come in different shapes and sizes. They may be formal constructs or informal groups based on cultural factors and relationships. They may be focused vertically on patient care or horizontally on mutual support at the provider level. They may cut across public and private sectors or be contained within a fixed health system. They may also be supported by policies and programs at the country level or developed organically. Not every network should be formally established, open to all members, and serve the dual purposes of patient care and provider support. Nor are informal, closed, or single-issue Networks of Care necessarily less effective. Regardless of the specific issues that networks may be optimized to address, they should all aim to achieve the following objectives.

- **Improve patient outcomes.** Networks should address both processes of care and structures of care. Handoffs can be the most dangerous part of patient care, and stronger networks help to reduce fragmentation in all the different types of handoffs (from prevention to seeking care, from care to treatment at higher levels, and from treatment to follow-up in terms of drugs/diagnostics).
- **Improve workforce satisfaction**. Overworked, disconnected, and under-resourced health workers face challenges in providing quality care. As a result, ongoing access and support from a group of peers and enhanced collaboration can overcome some of these challenges.
- **Enhance resiliency of the system**. Networks should support the systems’ ability to maintain (and even improve) services in the face of unexpected challenges. Having built-in redundancies through networks, as well as the ability to learn from peers dealing with the same challenges, enables this resiliency.

The key is to nurture and optimize Networks of Care to maximize the benefits we describe and to create networks that have the flexibility to adapt when needed but also have the precision to track a single patient through the course of a health condition to ensure high-quality care.

We call for a global commission to further build and develop the concept of Networks of Care in line with the framework we outline and with particular attention to understanding what tools exist and can be leveraged to support Networks of Care. We know digital solutions exist, including online meetings, communities of practice, and electronic medical records, but tools alone are insufficient to realize the full benefits of Networks of Care.

A global commission can provide direction on how to ensure a shared understanding of care and handoff protocols across providers and networks, what best facilitates clear lines of communication and engagement, what motivates providers to actively participate in different types of networks, how we can best incentivize and cultivate these motivations, and how network members can conduct and benefit from regular reviews of what is and isn’t working.
